# Short Assessment of Health Literacy (SAHL) in Portugal: development and validation of a self-administered tool

**DOI:** 10.1017/S1463423618000087

**Published:** 2018-02-15

**Authors:** Carla Pires, Pedro Rosa, Marina Vigário, Afonso Cavaco

**Affiliations:** 1 Researcher, Department of Social Pharmacy, Faculty of Pharmacy, University of Lisbon, Lisbon, Portugal; 2 Auxiliary Professor, School of Psychology and Life Sciences, Lusophone University of Humanities and Technologies, Lisbon, Portugal; 3 Cognition and People-Centric Computing Laboratories (COPELABS), ULHT, Lisbon, Portugal; 4 Instituto Universitário de Lisboa (ISCTE-IUL), Cis-IUL, Lisbon, Portugal; 5 Grupo Internacional de Investigación Neuro-Conductual (GIINCO), Barranquilla, Colombia; 6 Associate Professor, School of Arts and Humanities, Centre of Linguistics of the University of Lisbon, University of Lisbon, Lisbon, Portugal; 7 Associate Professor, Department of Social Pharmacy, Faculty of Pharmacy, Research Institute for Medicines and Pharmaceutical Sciences, University of Lisbon, Lisbon, Portugal

**Keywords:** health literacy, SAHL, self-administered questionnaire, short assessment tools, validation methods

## Abstract

The goal of this study was to adapt, improve and validate a short, self-administered health literacy assessment tool for European Portuguese-speaking adults. Health literacy tools are of great importance to health authorities and professionals, as low or inadequate health literacy, that is, a limited capacity to handle health-related information, is associated with higher morbidity and mortality. The 18-item Short Assessment of Health Literacy for Brazilian Portuguese-speaking adults (SAHLPA-18) was adapted into European Portuguese. The European Portuguese tool (SAHLPA-23) includes five additional items. The SAHLPA-23 was tested in a convenience sample of 503 participants from two Portuguese regions. Socio-demographic data, literacy and cognitive indicators were collected. Participants also completed a questionnaire on comprehension of written health materials. Construct validity was assessed through correlations between SAHLPA-23 scores and education, literacy, and cognitive variables and score on the comprehension questionnaire. The psychometric properties of the new tool were compared with those of the SAHLPA-18. The mean SAHLPA-18 and SAHLPA-23 scores were 13.9 (77.2%; SD=2.9) and 18.3 (79.6%; SD=3.8), respectively. Both tools showed adequate reliability (Cronbach’s *α*>0.7). SAHLPA-23 was more highly correlated with all study variables than SAHLPA-18. Although both instruments displayed acceptable discriminative power, SAHLPA-23 had better accuracy than SAHLPA-18 (DeLong’s method: ΔAUC=0.09, *Z*=3.36; *P*<0.001). The SAHLPA-23 is an independent, feasible and innovative tool for estimation of health literacy in the Portuguese adult population.

The concept of literacy has evolved over recent decades. In the late 20th century literacy was mainly understood in terms of reading and writing skills, but the concept has been extended and now includes the ability to interpret texts, perform calculations and participate actively in society (Organization for Economic Co-Operation and Development and Statistics Canada, 2011; Sorensen *et al*., 2012). As one would expect, there is wide evidence of a positive correlation between education and literacy, although less educated individuals may also be capable of executing complex literacy tasks as a result of regular reading and writing (Benavente *et al*., 1996; Sorensen *et al*., 2012).

As patients should be able to understand and manage relevant health information, literacy is an important issue in healthcare (McCray, 2005; Barber *et al*., 2009; Chinn, 2011; Mårtensson and Hensing, 2012; Sykes *et al*., 2013). Health literacy may be defined as the ability to obtain, process and understand information about health and health services as necessary to make appropriate health decisions (Sorensen *et al*., 2012: 3).

Studies of health literacy are relevant to health authorities worldwide, since low health literacy is associated with a higher morbidity and mortality and lower treatment adherence; it is also associated with a lower capacity for making health decisions, managing health information and using medication (Dewalt *et al*., 2004; Berkman *et al*., 2011; Zhang *et al*., 2014). Previous research has shown that low health literacy is more prevalent amongst older people, people with low income and people with few years of education, so these factors contribute to health inequalities (Zamora and Clingerman, 2011; Bostock and Steptoe, 2012; Raynor, 2012; Findley, 2015; Greenhalgh, 2015). Even in developed countries a high proportion of the population has difficulty managing health information. For instance, the European Health Literacy Project (HLS-EU Project, 2009–2012), which collected data from 8000 participants in eight European countries (Austria, Bulgaria, Germany, Greece, Ireland, the Netherlands, Poland and Spain), revealed limited health literacy skills in 47.5% of the people evaluated (HLS-EU, 2009–2012). The HLS-EU-Questionnaire (HLS-EU-Q) comprises 47 self-report items, to which responses are given using a four-point scale. This questionnaire was used to measure participants’ perception of the difficulty of various health-related tasks. Threshold values were defined, and the scores classified into four health literacy categories: ‘inadequate’, ‘problematic’, ‘sufficient’ and ‘excellent’. Data collected with the HLS-EU-Q in Portugal showed low rates of health literacy: 61.5% of the 1001 participants were categorised as having insufficient health literacy (HLS-EU Project, 2014). Results from another country, mentioned in the report of the National Assessment of Health Literacy of America’s Adults, indicated that 35% of the 18 186 adults assessed had unsatisfactory health literacy skills (National Center for Education Statistics and US Department of Education, 2003). This study categorised participants using a set of performance levels (Below Basic, Basic, Intermediate and Proficient) on the basis of performance on prose, document and quantitative tasks. Prose tasks were related to the knowledge and skills needed to search, comprehend and use information from continuous texts; document tasks tapped the ability to search, comprehend and use information from non-continuous texts in various formats; and quantitative tasks were related to the identification and performance of computations, either alone or sequentially, using numbers embedded in printed material (National Center for Education Statistics and US Department of Education, 2003). The Australian Adult Literacy and Life Skills Survey reported that 59% of the 15 105 participants had inadequate health literacy skills (Australian Bureau of Statistics, 2006). The survey evaluated knowledge and skills in four literacy domains: prose, document, numeracy and problem solving. A fifth indicator, health literacy, was also evaluated (Australian Bureau of Statistics, 2006).

Various tools and methodological approaches have been used to evaluate health literacy (Lee *et al*., 2010; Collins *et al*., 2012; Haun *et al*., 2014; Duell *et al*., 2015), such as:

- The Rapid Estimate of Adult Literacy in Medicine (REALM, 1991): participants are required to read and recognise health words (66 items, 3 min).
- The Test of Functional Health Literacy in Adults (TOFHLA, 1995): participants are required to perform a modified cloze procedure (health-related information) (67 items, 18–22 min).
- The Newest Vital Sign (NVS, 2005): participants are required to answer comprehension questions about the nutrition label for an ice cream (six items, around 6 min).
- The Short Assessment of Health Literacy - Spanish and English versions (SAHL-S; SAHL-E, 2010): participants are required to read and recognise health words (18 items, 2-3 min).

As mentioned before, health literacy tools can be prose, oral or numeracy tasks, and may include self-evaluation items, which may influence their accuracy (Altin *et al*., 2014; Morrison *et al*., 2014). Several limitations of the existing health literacy tools have been identified, for example, the length of time and cost of administering them; in addition, these tools are likely to be unsuitable for routine use in healthcare consultations. The patients’ screening of health literacy is preferentially recommended in the first clinical appointment to ensure that health-related information is comprehensible for all subjects through simple, intelligible and effective communication (Altin *et al*., 2014; Hersh *et al*., 2015). The evaluation of other potentially relevant social aspects, such as participants’ beliefs and behaviours is also considered pertinent when designing health literacy tools (Guzys *et al*., 2015).

There are only a few published short, self-administered literacy tools, for example, the REALM-Short Form and the Medical Term Recognition Test (METER) (Lee *et al*., 2010; Apolinario *et al*., 2012; Collins *et al*., 2012; Haun *et al*., 2014; Paiva *et al*., 2014; Duell *et al*., 2015; Guzys *et al*., 2015). Also, there are a few short health literacy tools specifically validated for use with European Portuguese speakers (Benavente *et al*., 1996; Soares, 2010; Paiva *et al*., 2014). For instance, Paiva *et al*. (2014) have validated a version of the short health literacy assessment tool METER for use in the Portuguese adult population. This self-administered tool is based on REALM and consists of a list of 40 English medical words and 30 non-words which sound like genuine medical terms. Respondents are required to identify the genuine medical words and scores are calculated as the sum of all the correctly identified words. The health literacy categories for the original METER are as follows: low (0–20), marginal (21–34) and functional (35–40) (Paiva *et al*., 2014). Two versions of SAHL, a 50-item version (SAHLPA-50) and a shorter 18-item version (SAHLPA-18) have recently been validated for use with Brazilian Portuguese-speaking older adults (Apolinario *et al*., 2012). The SAHLPA items consist of cards with a medical term printed in bold at the top and two association words at bottom, only one of which is associated with the medical term printed above. First, the respondent is required to read the medical term aloud, then the interviewer reads the two association words and finally the respondent has to choose and read aloud the correct response. Answers are only judged correct if the respondent both chooses the correct word and pronounces it correctly. The SAHLPA-50 takes 3-6 min to complete and the SAHLPA-18 1–2 min (Lee *et al*., 2010; Apolinario *et al*., 2012). The choice of a health literacy tool for any given situation will depend on several factors, such as the required accuracy and time available for the assessment (Collins *et al*., 2012; Haun *et al*., 2014; Duell *et al*., 2015). One of the advantages of the SAHL is that it is particularly suitable for evaluating individuals with low health literacy. It has also demonstrated higher reliability than other health literacy indices (Lee *et al*., 2010; Altin *et al*., 2014). The Apolinario *et al*. (2012) short version of SAHL is smaller than METER (18 versus 40 items) and this is possible because the SAHL procedure implies respondents’ semantic understanding of medical terms, rather than just requiring them to distinguish medical terms from non-words (Lee *et al*., 2010; Apolinario *et al*., 2012; Altin *et al*., 2014; Paiva *et al*., 2014). The SAHL has been validated in Brazil, but this does not guarantee that it is suitable for assessing the health literacy of the Portuguese population, as there are linguistic differences between Brazilian and European Portuguese as well as social and cultural differences between the two countries (Costa *et al*., 2007; Soares, 2010; Apolinario *et al*., 2012).

As mentioned previously the HLS-EU project (2014) showed that the Portuguese population has a low rate of health literacy and this suggests there is a real need to increase the range of health literacy tools available at national level. Health literacy tools that are simpler and cheaper to administer are more suitable for used in healthcare settings (Apolinario *et al*., 2012; Paiva *et al*., 2014). The Portuguese government has recently created a national Programme of Education for Health, Literacy and Self-Care (Law – *Despacho* no. 3618-A/2016); this demonstrates that the decision-makers have recognised the population’s low level of health literacy and the need to improve it. In addition, Portuguese, taking the European, Brazilian, African and Asian varieties together, is the world’ seventh most commonly spoken language (with around 200 million of speakers) (Nations Online Project, 2017), which provides another reason to develop Portuguese-language health literacy tools.

The evidence and information reviewed above, together with the fact that public health sector resources remain very limited in Portugal (Fernandes and Nunes, 2016) suggest that there is an urgent need for development of short health literacy tools suitable for the Portuguese population. Hence, the objectives of this study were to adapt, improve and validate a short, self-administered version of SAHL for European Portuguese-speaking adults.

## Methods

We present two versions of the SAHL, the original SAHLPA-18 adapted from Apolinario *et al*. (2012) and a newly developed adaptation, the SAHLPA-23. This section describes the procedure used to adapt the SAHLPA, the method of administration and the scoring of the new tool (SAHLPA-23). The recruitment and inclusion criteria and the protocol for a brief cognitive evaluation are also described. A comprehension questionnaire on a health document was administered and direct and indirect literacy indices were defined and used to assess the concurrent validity of the new tool. Finally, the statistical analysis procedures are described.

### Short health literacy tool: SAHLPA-18 and SAHLPA-23

The SAHLPA-23 (Table 1) consists of the recently validated SAHLPA-18 for Brazilian Portuguese-speaking older adults (Apolinario *et al*., 2012) plus an additional five items dealing with medication-related literacy issues (SAHLPA-23). The inclusion of five new items was expected to improve the psychometric proprieties of the tool, in particular its specificity (Pander Maat *et al*., 2014).

**Table 1:** Tested tool: Short Assessment of Health Literacy for Portuguese-Speaking Adults (SAHLPA-23)

Term (Portuguese/English)	Answer 1 (Portuguese/English)	Answer 2 (Portuguese/English)	Answer 3 (Portuguese/English)
*Próstata*	______ *glândula*	______ *circulação*	______ *não sei*
Prostate	______ gland	______ circulation	______ don’t know
*Substância ativa* ^a^	______ *agente secundário*	______ *componente principal*	______ *não sei*
Active substance	______ secondary component	______ principal component	______ don’t know
*Efeitos secundários* ^a^	______ *reações inesperadas*	______ *ações esperadas*	______ *não sei*
Drug adverse reactions	______ unexpected reactions	______ expected actions	______ don’t know
*Menstrual*	______ *mensal*	______ *diário*	______ *não sei*
Menstrual	______ monthly	______ *daily*	______ don’t know
*Cafeína*	______ *energia*	______ *água*	______ *não sei*
Caffeine	______ energy	______ water	______ don’t know
*Osteoporose*	______ *osso*	______ *músculo*	______ *não sei*
Osteoporosis	______ bone	______ muscle	______ don’t know
*Incesto*	______ *família*	______ *vizinhos*	______ *não sei*
Incest	______ family	______ neighbours	______ don’t know
*Testículo*	______ *óvulo*	______ *esperma*	______ *não sei*
Testicle	______ ovule	______ sperm	______ don’t know
*Rectal*	______ *regador*	______ *sanita*	______ *não sei*
Rectal	______ watering can	______ toilet	______ don’t know
*Anormal*	______ *diferente*	______ *similar*	______ *não sei*
Abnormal	______ different	______ similar	______ don’t know
*Aborto*	______ *perda*	______ *casamento*	______ *não sei*
Miscarriage	______ loss	______ marriage	______ don’t know
*Excipiente* ^a^	______ *agente principal*	______ *componente secundário*	______ *não sei*
Excipient	______ principal component	______ secondary component	______ don’t know
*Icterícia*	______ *amarelo*	______ *branco*	______ *não sei*
Jaundice	______ yellow	______ white	______ don’t know
*Papanicolau*	______ *teste*	______ *vacina*	______ *não sei*
Pap	______ test	______ vaccine	______ don’t know
*Apêndice*	______ *raspar*	______ *dor*	______ *não sei*
Appendix	______ scratch	______ pain	______ don’t know
*Fertilidade* ^a^	______ *reprodução*	______ *ação*	______ *não sei*
Fertility	______ reproduction	______ action	______ don’t know
*Comportamento*	______ *pensamento*	______ *conduta*	______ *não sei*
Behaviour	______ thought	______ conduct	______ don’t know
*Hemorroida*	______ *veia*	______ *coração*	______ *não sei*
Haemorrhoid	______ vein	______ heart	______ don’t know
*Colite*	______ *intestino*	______ *bexiga*	______ *não sei*
Colitis	______ bowel	______ bladder	______ don’t know
*Precauções* ^a^	______ *cuidados*	______ *contraindicações*	______ *não sei*
Precautions	______ care	______ contraindications	______ don’t know
*Vesícula biliar*	______ *artéria*	______ *órgão*	______ *não sei*
Gallbladder	______ artery	______ organ	______ don’t know
*Convulsão*	______ *tontura*	______ *calma*	______ *não sei*
Seizure	______ dizzy	______ calm	______ don’t know
*Artrite*	______ *estomâgo*	______ *articulação*	______ *não sei*
Arthritis	______ stomach	______ articulation	______ don’t know

a The five new items specifically selected from the Quality Review of Documents template (European Commission, 2009). SAHLPA-23 corresponds to SAHLPA-18 (Apolinario *et al*., 2012) plus the five new items. SAHLPA-23 was the only tool administered. The words in italics correspond to those presented, written in Portuguese.

The original 18-item tool was validated in a sample of participants with limited education (74.4% of the sample had ⩽seven years of schooling; Apolinario *et al*., 2012), making it suitable for used in the Portuguese population, which has limited health literacy (Portuguese Institute of Statistic, 2011; HLS-EU Project, 2014). Each SAHPLA-23 item requires the respondent to select, from two association words, the word which best describes the target medical term (eg, ‘prostate’ may be correctly described as a ‘gland’ or incorrectly described as ‘circulation’). The respondent may also choose a third response option: ‘do not know’ (Table 1). Two pharmacists and one linguist were involved in adapting the SAHLPA-18; in particular they were, required to check possible linguistic differences between European and Brazilian varieties of Portuguese (Lee *et al*., 2010; Apolinario *et al*., 2012). Few changes were required, perhaps due to the technical nature of the items.

### Additional items: SAHPLA-23 and scoring

The introduction of new items during adaptation of health literacy tools has been reported in prior studies (Lee *et al*., 2006; Richman *et al*., 2007). The five new items were selected from the Quality Review of Documents template for European package leaflets (PLs), based on their high frequency in written information designed for patients and consensus amongst the research team. All the new target terms belong to the healthcare domain and are widely used at a national and international level (European Medicine Agency, 2016). They are: ‘active ingredient’ (*substância ativa*), ‘adverse reactions’ (*efeitos secundários*), ‘excipient’ (*excipiente*), ‘fertility’ (*fertilidade*) and ‘precautions’ (*precauções*). Two of the five new items were deliberately designed with two-word targets in European Portuguese (‘active ingredient’ and ‘adverse reactions’) as it was thought that longer targets would provide better discrimination between levels of health literacy. The positioning of the five additional items in the tool is random (Table 1). All the correct responses score 1 point and all other responses score 0 points, thus SAHLPA-18 and SAHLPA-23 scores range between 0 and 18 points and 0 and 23 points, respectively. Scores on the items included in the original version of the tool (SAHLPA-18) were compared with the scores of SAHLPA-23 in order to determine which offered the better measure of the construct of health literacy.

### Collection of socio-demographic data and comprehension questionnaires

A set of additional self-administered tools was used to collect socio-demographic data (ie, age, gender, income level, education, employment and civil status) and assess comprehension of health-related documents. Comprehension was evaluated with two questionnaires on the comprehension of PLs of medicinal products. These last two questionnaires were developed in accordance with the European guidelines on medicine PLs clarity and comprised eight questions (Appendix 1) (European Commission, 2009; Portuguese Institute of Statistics, 2011). They were scored as follows: incorrect answer: 0 points; partially correct answer containing some incorrect information: 1 point; partially correct answer without any incorrect information: 2 points; correct answer: 3 points; thus the maximum score was total score for each was 24 points. These instruments, as well as the structured protocol for evaluation of PL comprehension, were developed and validated in a previous study (Pires *et al*., 2016). Scores on the comprehension questionnaires, as well the direct and indirect literacy indices (described in the next section) were necessary for the validation of the health literacy tool (Apolinario *et al*., 2012).

### Direct and indirect literacy indexes

The direct literacy index was composed of the following literacy tasks:

- The spelling of the words *carro* (car), *vaso* (vase), *bola* (ball), lápis (pencil) and *relógio* (watch) (maximum score: 5 points).
- The mental calculus task: 100−7(maximum score=1 point), plus two additional calculation tasks (maximum score=2 points). The two additional calculation tasks were as follows: a sum followed by a subtraction to calculate how much money respondents will get back in a purchase and a division to calculate the price of a certain amount of a product. These last two calculation tasks were adapted from the last national Portuguese literacy study (Benavente *et al*., 1996). The maximum score on the direct index was 8 points.

The indirect literacy index was based on participants’ declarations about their reading and writing habits. It included closed questions about how often participants read material in the following categories: (1) books (six per year, five or six per year, three or four per year; one or two per year, rarely and never; maximum score: 5 points); and (2) magazines or journals (daily, at least once a month, at least once a week, rarely and never; maximum score: 4 points). Participants’ writing habits were also assessed, using questions about how often they performed simple writing tasks (eg, writing phone messages). The response options were as follows: daily, at least once a week, at least once a month, rarely and never (maximum score: 4 points). These questions were also adapted from the last national Portuguese literacy study 1995, reported by Benavente *et al*. (1996). The maximum total score on the indirect literacy index was 13 points.

### The cognitive index

Before administering the SAHLPA-23 and the study questionnaires a brief screening test was administered to detect any cognitive problems (eg, dementia) that might affect participants’ judgement and reasoning capacity. The test consisted of the following questions and simple tasks: (1) ‘What month is it?’ (2) ‘Where are we (what city, town, or village)?’ (3) ‘The researcher is about to say three words in loud voice: please write them down (the words were car, vase and ball),’ (4) ‘How much is 100 minus 7?’ (5) The researcher shows the participant a pencil and asks him or her to write down the name of the object, (6) The researcher shows the participant a watch and asks him or her to write the name of the object and (7) ‘Write down the three words that the researcher said in loud voice a few minutes ago, again.’ These questions and tasks were based on the full version of the Mini-Mental State Examination (Folstein *et al*., 1975; Grady, 2008) and scored as follows: 1 point each for correct responses to questions 1, 2 and 4-6 (0 for incorrect responses) and 1 point for each correct word on questions 3 and 7 (maximum of 3 points per question). As the aim was to check participants’ ability to recognise and write words pronounced in loud voice spelling errors were not taken into account in scoring the cognitive index; however, they were taken into account when calculating the direct literacy index. We included calculation of 100−7 because this task is not supposed to be difficult, especially for the more literate participants. The maximum score on the cognitive index was 11 points. Importantly, our sample had a mean score of 10.8 in the cognitive index, indicating that the participants’ were likely to have sufficient cognitive capacity to complete the other assessments.

### Recruitment and selection of the participants

The participants were a convenience sample recruited from the Lisbon and Tagus Valley and Central Portugal regions between August and December 2014. These administrative regions differ with respect to several characteristics: the former is urban, coastal and has a high population, whereas the latter is rural, inland and has a low population (Pires *et al*., 2016). An email invitation was sent to various city-hall services, military institutions, fire-fighting departments, public cleaning services, parish centres, residential and nursing homes, and education institutions from the two regions. The inclusion criteria were as follows: age of at least 18 years (the age of legal majority in Portugal; Public Ministry, 1966), willingness to participate, having Portuguese as native language and being able to read and write. A sample of 503 participants was recruited, 49.3% from Lisbon. The required sample size was calculated using the formula given by Krejcie and Morgan (1970) (Altin *et al*., 2014) and the population data from the last Portuguese census (Krejcie and Morgan, 1970). A detailed explanation of the calculation of the sample size can be found in an earlier study (Pires *et al*., 2016).

### Administration of the short health literacy tool

The tool (SAHLPA-23; see Table 1) was designed to be self-administered, although participants were given oral instructions on how complete it. It was distributed to all participants and completion was overseen by a researcher. Immediately after the questionnaires had been distributed participants were instructed to read the term written in the first column and to mark the option that was most closely related to it, or the ‘do not know’ option with a cross (Table 1). Participants were told that they could ask for clarification if they were unsure about how to complete the questionnaire, but the overwhelming majority (99%) had no problems. Participants were given a maximum of 15 min to complete the SAHLPA-23. This time period was defined based on the results of a pilot test with five elderly participants with limited education. Only 11 participants (5.9%) declared to have spent the 15 min, thus the period allowed was deemed adequate to allow respondents to complete the tool without rushing. The study materials were randomly distributed to the participants to avoid an uneven use of time.

### Statistical analysis

Descriptive statistics were calculated. As the percentage of missing values was below 3% no imputation method was applied. A multi-step validation of the SAHLPA-23 was carried out as follows: (1) exploratory factor analysis (EFA) was carried out [Kaiser–Meyer–Olkin Measure (KMO) of sampling and adequacy and the Bartlett’s Test of Sphericity]. Principal component factor analysis with oblique rotation (Direct Oblimin with the delta parameter equal to 0) was conducted based on eigenvalues values (Kaiser’s criterion: eigenvalues ˃1 and the scree plot as the extraction strategy) (Pedhazur and Schmelkin, 1991). (2) The reliability of both the SAHLPA-18 and SAHLPA-23 was expressed as Cronbach’s *α*0.(3) The discriminatory ability of both tests was examined using receiver operating characteristic (ROC) curves and area under the curve (AUC). The optimal cut-off point was defined as the maximum value of the Youden index, calculated as: *J*=max [sensitivity *c*+specificity *c*–1], where *c* represents all possible criterion values (Youden, 1950). The difference between AUCs was assessed using DeLong’s non-parametric approach (DeLong *et al*., 1988).

A cut-off score for adequate health literacy was identified using ROCs for the established literacy tasks. This procedure resulted in exclusion of some items and the definition of a cut-off score for distinguishing between adequate and inadequate health literacy. (4) Spearman’s rank test was used to assess correlations between score on the new short health literacy tool (SAHLPA-23) and the construct variables. Construct validity was evaluated by calculating correlations between SAHLPA-23 scores and the following variables: years of schooling, cognitive index, literacy tasks, reading and writing habits, score on the comprehension questionnaire, and the socio-demographic variables. The Statistical Package for Social Sciences (SPSS) version 22 (IBM SPSS Inc., Chicago, IL, USA) was used for all analyses except for ROC analyses, which were conducted using MedCalc version 14.8 (MedCalc Software, Mariakerke, Belgium). All statistical tests were two-tailed (*α* level<0.05).

## Results

Here, we present the socio-demographic data, data from the direct and indirect literacy indices, cognitive assessment and the scores of SAHLPA-23 and SAHLPA-18. We also compare the psychometric properties of SAHLPA-23 and SAHLPA-18 using EFA, Cronbach’s *α*, tests of construct validity, Spearman’s rank test, outliers, ROC curves and AUCs.

### Socio-demographic data

The final sample consisted of 484 adults as 19 participants from the original sample had to be excluded for statistical reasons. The participants were mainly from city-hall services (26.4%), the military (21.3%) and university undergraduates (non-biomedical studies) (19.9%). The remaining participants were evenly distributed across the other institutions. Overall, 49.3% of the participants were from Lisbon and Tagus Valley, 53.1% (*n*=256) were male, with a mean age of 38.7 year (SD=18.2, range 18-88) and an average of 10.3 years (SD=4.8) of schooling (Table 2). Table 3 shows the scores on the study variables.

**Table 2:** Characteristics of the participants (*N*=484)

Variables	n	%
Gender		
Male	256	53.1
Female	226	46.9
Age (years)		
18–30	218	45.0
31–40	67	13.8
41–50	69	14.3
51–60	66	13.6
>60	64	13.2
Income per month		
None	85	17.6
<485 euros	71	14.7
485–970 euros	263	54.3
>970 euros	65	13.4
Labour situation		
Employed	272	57
Retired	61	12.7
Other^a^	151	30.3
Civil status		
Single	243	50.2
Married	159	32.9
Divorced	28	5.8
Widowed	33	6.8
Unmarried partnership	19	3.9
Separated	2	0.4
Schooling (years)^b^		
1–6	148	30.6
7–12	180	37.2
>12	156	32.2

n=number of participants.

a The sex of two participants was not registered.

b The average number of years of schooling by each one of the three education groups were as follows: average=4.2, SD=1.3 (⩽6); average=10.8, SD=1.4 (7–12); and average=15.4, SD=1.4 (>12 years of schooling).

**Table 3:** Mean scores and standard deviations for the study variables

	Mean	SD
SAHLPA-18	13.9	2.9
SAHLPA-23	18.3	3.8
Cognitive assessment	10.5	1
Direct literacy index	6.2	1.6
Indirect literacy index	11.8	3.3
Comprehension questionnaire	15.1	7.6

SAHLPA-18 and SAHLPA-23=Short Assessment of Health Literacy for Portuguese-Speaking Adults with 18 and 23 items, respectively. SAHLPA-23 was the only administered tool, that is, SAHLPA-18 is referent to a separate analyse comprising the results for the items of the original version of Apolinario *et al*. (2012).

### EFA of SAHLPA-23 and SAHLPA-18

The SAHLPA-23 data yielded a good KMO score (0.89) and Bartlett’s sphericity test was significant [*χ* ^2^(253)=2764.4, *P*<0.001], indicating that the correlation matrix was suitable for factor analysis. According to the ratio-of-first-to-second-eigenvalues-greater-than-three rule (Slocum-Gorit and Zumbo, 2011) the SAHLPA-23 displayed a one-dimensional structure (5.97/1.78=3.36).

The SAHLPA-18 was subjected to a similar factor analysis. The data displayed a KMO score (0.87) and Bartlett’s sphericity test was significant [*χ* ^2^(153)=1963.6, *P*<0.001], indicating that the correlation matrix was suitable for factor analysis. As with the SAHLPA-23, the ratio-of-first-to-second-eigenvalues-greater-than-three rule also indicated that a one-dimensional structure (4.97/1.52=3.32). Visual inspection of the scree plots for both instruments also revealed a multidimensional factor structure (Appendix 2).

### Cronbach’s *α*: SAHLPA-23 and SAHLPA-18

Internal consistency was estimated via Cronbach’s *α* (Appendix 3); 0.70 was regarded as the acceptable minimum (Tavakol and Dennick, 2011). The corrected item-total correlations and *α*-if-item-deleted values were also examined for SAHLPA-23. There were only two items where removal increased Cronbach’s *α* (items 15 and 19, ie, none of the five new items) and the resulting improvements in Cronbach’s *α* were minimal (<0.01) so all the items were retained. Cronbach’s *α* was 0.81 for the SAHLPA-18, indicating good reliability (Appendix 3).

### Construct validity and Spearman’s rank test

As the data were not normally distributed Spearman’s rank order correlations were used to assess construct validity. The SAHLPA-23 was positively correlated (all *P*’s<0.05) with schooling [*r*_s_(482)=0.537], the cognitive index [*r*_s_(482)=0.374], the comprehension questionnaire [*r*_s_(482)=0.561], direct measures of literacy [*r*_s_(482)=0.308] and indirect measures of literacy [*r*_s_(482)=0.234], suggesting that the SAHLPA-23 had good construct validity. The SAHLPA-23 was more highly correlated with all relevant study variables, except the direct and indirect measures of literacy, than the SAHLPA-18 (Table 4).

**Table 4:** Spearman’s correlation coefficients between the SAHLPA-23/SAHLPA-18 and study variables

Study variables	SAHLPA-23	SAHLPA-18
Schooling^a^	0.472***	0.537***
Cognitive assessment	0.177***	0.234***
Comprehension questionnaire	0.420***	0.561***
Direct measures of literacy	0.439***	0.308***
Indirect measures of literacy	0.308***	0.234***

SAHLPA-18 and SAHLPA-23=Short Assessment of Health Literacy for Portuguese-Speaking Adults with 18 and 23 items, respectively. The study variables are the selected variables for the validation procedure. SAHLPA-23 was the only administered tool, that is, SAHLPA-18 is referent to a separate analyse comprising the results for the items of the original version of Apolinario *et al*. (2012).

a Three education groups were considered: ⩽6, 7–12 and >12 years of schooling.

***P<0.001 (*n*=484).

### Spearman’s rank test and Fisher *r*-to-*z* transformation

Fisher *r*-to-*z* transformation was used to assess the significance of the difference between the correlation coefficients relating the SAHLPA-23 and SAHLPA-18 to variables of interest. The Spearman’s correlation coefficient for association with comprehension questionnaire score was larger in the case of the SAHLPA-23 than the SAHLPA-18 (*Z*=2.89, *P*=0.003). Similarly, the association with the direct literacy index was larger in the case of the SAHLPA-23 than the SAHLPA-18 (*Z*=2.26, *P*=0.023). The rest of the variables were similarly associated with both instruments (*P*>0.05).

### Outliers, ROC curves and AUCs

In accordance with classical data reduction procedures (Bain and Engelhardt, 1992) all the SAHLPA-23 scores more than 2 SD above or below the mean of each independent group^1^ were considered outliers. Altogether, 19 outliers (3.78% of the sample) were removed; eight from the adequate health literacy group and 11 from the inadequate health literacy group. Thus, the final sample comprised 484 participants.

**Figure 1:**
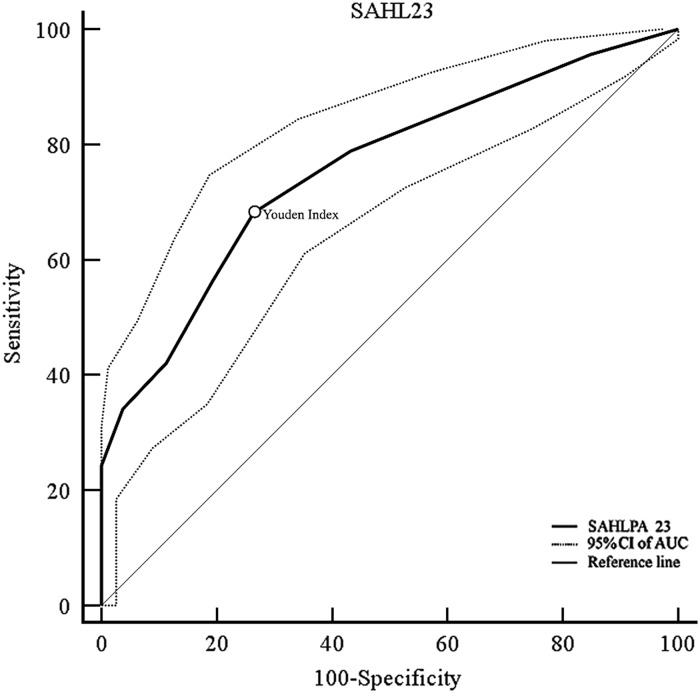
Receiver operating characteristic curve for detection of inadequate health literacy (*N*=484). SAHL=Short Assessment of Health Literacy; SAHLPA=Short Assessment of Health Literacy for Brazilian Portuguese-speaking adults; CI=confidence interval; AUC=area under the curve.

Participants scoring <20 (80%) (maximum score=24) on our structured protocol for comprehension of PLs were defined has having inadequate health literacy. This cut-off score was based on the recommendations of the European Medicines Agency as expressed in the *Guidelines on the readability of the labelling and package leaflet of medicinal products for human use* (European Commission, 2009). Using this cut-off 310 (61.6%) participants were classed as having inadequate health literacy and 193 (38.4%) were classed as having adequate health literacy. As can be seen from Figure 1, the SAHLPA-23 AUC for detection of inadequate health literacy was 0.76 (95% confidence interval [0.73; 0.80], *P*<0.001), which suggests the tool is a moderately accurate method of detecting inadequate health literacy. The recommended minimum AUC for diagnostic tests is 0.75 (Larner, 2015).

The best SAHLPA-23 cut-off value for detection of inadequate heath literacy was ⩽19 (Youden’s *J*=0.42; sensitivity=68.2%; specificity=73.5%) (Appendix 4). This cut-off value represents ~83% of the maximum possible score and resulted in 53% participants (257 out of 484) being classified as having inadequate health literacy. The 50-item Brazilian version of SAHLPA has a similar optimal cut-off, 42 (84% of the maximum possible score) (Apolinario *et al*., 2012).

### Comparison of AUCs: DeLong’s method

**Figure 2:**
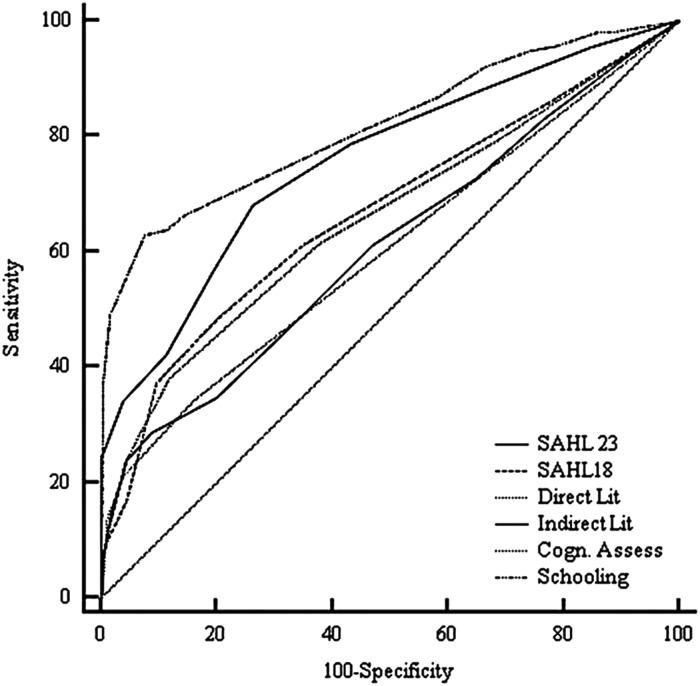
Receiver operating characteristic curves for detection of inadequate health literacy between SAHLPA-23 and other variables (*N*=484). SAHL=Short Assessment of Health Literacy; SAHLPA=Short Assessment of Health Literacy for Brazilian Portuguese-speaking adults.

Comparison of the AUCs using DeLong’s method revealed that the SAHLPA-23 was a more accurate method of discriminating levels of health literacy than the SAHLPA-18, ΔAUC=0.09, *Z*=3.36, *P*<0.001. The SAHLPA-23 also had a larger AUC than the direct measure of literacy, ΔAUC=0.10, *Z*=3.40, *P*<0.001 and the indirect measure of literacy, ΔAUC=0.15, *Z*=4.70, *P*<0.001 (Figure 2).

Years of formal schooling (<7; 7-12; >12) had the highest AUC, which was higher than the AUC for the SAHLPA-23 (*Z*=2.49, *P*=0.02). Comparisons of the ROC curves for SAHLPA-23 and other instruments are shown in Table 5.

**Table 5:** Pairwise comparison of area under the curves (AUCs) between SAHLPA-23 and other variables (*N*=484)

Pairwise comparison	ΔAUC	SE	95% CI	*Z*
SAHLPA-23–SAHLPA-18	0.09	0.03	0.04–0.15	3.3***
SAHLPA-23–Ind_literacy	0.06	0.03	0.00–0.12	4.79***
SAHLPA-23–Dir_literacy	0.02	0.03	−0.03–0.07	3.40***
SAHLPA-23–Cog_Assess	0.03	0.03	−0.02–0.09	5.82***
SAHLPA-23–Schooling	0.06	0.02	0.00–0.10	2.48*

SAHLPA-23=Short Assessment of Health Literacy for Portuguese-Speaking Adults with 23 items. *P<0.05; ***P<0.001.

## Discussion

The aim of this study was to assess the suitability of the SAHLPA-23 and SAHLPA-18 for use as brief, self-administered tools for evaluating health literacy in the adult Portuguese population. Both SAHLPA-23 and SAHLPA-18 showed suitable psychometric properties: one-dimensional factor structure, Cronbach’s *α* >0.7, mean-corrected item-total correlation >0.7 and high positive correlations with convergent variables. Moreover, the results are in line with the findings of Apolinario *et al*. (2012). Although both tools showed adequate reliability and good construct validity, the SAHLPA-23 is a better method of assessing health literacy as it discriminates more accurately between inadequate and adequate levels of health literacy (ie, has a higher AUC). It was confirmed that the addition of five new items to the SAHLPA-18 was advantageous.

### Administration of SAHLPA-23

The SAHLPA-23 appears suitable for self-administration as most participants had no questions about the completion procedure and took less than 15 min to complete the assessment; furthermore no responses had to be excluded due to failure to comply with instructions. The fact that the tool is self-administered may contribute to the length of time it takes to complete, which is somewhat longer than for other tools, such as SAHL-S and -E, which only requires 2–3 min to complete (Lee *et al*., 2010; Haun *et al*., 2014). In comparison with previous versions of the same tool (Apolinario *et al*., 2012) the SAHLPA-23 has the advantage that administration does not depend on the direct involvement of a health professional/researcher. Participants were required to mark their chosen response option with a cross rather than reading it aloud (Lee *et al*., 2010; Apolinario *et al*., 2012) as a result recordings and transcriptions of the oral answers were not required. It is worth noting that recordings of oral answers should be checked by linguistic experts to ensure that the medical terms are pronounced correctly, but we have not found reference to such a procedure in reports on the other versions of this tool (Lee *et al*., 2010; Apolinario *et al*., 2012).

### Health literacy findings from SAHLPA-23

Around half the participants (53%) were classed as having inadequate health literacy with the SAHLPA-23. This figure is slightly higher than that obtained in the last Portuguese health literacy study, in which only 38.5% of the 1001 participants were classified has having adequate or excellent health literacy (HLS-EU Project, 2014). This discrepancy may be due to the difference between the tools used (a questionnaire versus a short health literacy tool). Importantly, a recent validation of NVS in the Portuguese population yielded almost identical findings to this study, with 54% of the participants classed as probably having inadequate health literacy (Soares, 2010). This suggests that the SAHLPA-23 may be positively correlated with other health literacy tools previously validated for use in the Portuguese population. These findings provide additional evidence that the SAHLPA-23 is suitable for screening health literacy in the Portuguese population.

### Direct and indirect indices of literacy

Given that the mean score on the indirect literacy index (closed questions on reading and writing habits) was higher than the mean score on the direct literacy index (numeracy tasks), it is likely some participants overestimated their reading and writing habits (Schmidt and Retelsdorf, 2016). Additionally, writing habits were evaluated based on self-reported frequency of performance of simple tasks (eg, writing phone messages), which may reduced the specificity of this variable as an index of health literacy.

### PL comprehension questionnaire

This variable contributed to support construct validity. The mean score for this prose task was low, with only around half of the participants scoring ⩾75% (Pires *et al*., 2016). This suggests that the documents on which it was based were too complex.

### Study strengths

In line with other studies (Collins *et al*., 2012; Altin *et al*., 2014), the following validation requisites were met:

- a valid construct was defined;
- Cronbach’s *α* was appropriate;
- a large sample was enrolled;
- ROC curves were calculated and analysis of the AUCs confirmed that the tools showed adequate discrimination
- a cut-off for discriminating between probable adequate and inadequate health literacy was defined;
- an appropriate number of questions was defined;
- the administration procedure has been documented;
- the method of calculating the score has been described;
- the study costs were low;
- the time needed to administer the tool was acceptable.

### Study limitations

To validate a new tool it is important to compare the results with those obtained from administration of a ‘gold standard’ assessment, for example, a previous validated tool such as NVS (Collins *et al*., 2012; Altin *et al*., 2014). This was not possible due time constraints and the difficulty of recruiting participants willing to complete both assessments. Additionally, the cognitive assessment needed to be more robust to eliminate the possibility that the sample included cognitively impaired participants; it would have been better to use a validated tool such as the ‘Mini-Mental State Examination’ (Folstein *et al*., 1975; Grady, 2008).

Previous analyses of health literacy (Guzys *et al*., 2015) have taken into account socio-demographic data and social context (eg, social habits and health beliefs) and this has been done in previous studies of health literacy and it is recommended practice to contextualise evaluation of health literacy by carrying out a concurrent social evaluation. It is also recommended that the evaluation include a more robust assessment of reading and writing habits (eg, asking participants for the titles of books they have read in the previous year or collecting writing samples).

Schooling was the best predictor of health literacy in our study, that is, the AUC for schooling was significantly higher than the AUC for SAHLPA-23. This result might or might not be replicated in other samples, as our sample was not statistically representative of the Portuguese population. This means that our results may not generalise to national level. Moreover our low- and medium-education groups had had 4 and 11-12 years of schooling, respectively, leaving a gap in the distribution of years of education. This is mainly due to historical changes in the length of compulsory education in Portugal, which has changed from four years (in 1960) to nine years (in 1986) and eventually 12 years (in 2009).^2^ The number of participants in medium-education group (7-12 years of education) with nine years of schooling may have been low because the sample was recruited from public institutions where posts usually require at least 12 years of schooling and often additional education.

### Practical implications

SAHLPA-23 may be particularly useful to Portuguese health professionals and researchers as a quick method of obtaining an indication of health literacy in clinical and research settings, in order to anticipate potential problems with health literacy and facilitate more effective communication. Where possible socio-demographic data, such as years of education, should be collect and analysed together with this literacy measure.

## Conclusion

The SAHLPA-23 is an independent, effective and innovative self-administered tool for discriminating between adequate and inadequate health literacy. The SAHLPA-23 is a self-administered tool, unlike previous versions of the tool.

## Figures and Tables

**Figure A1:**
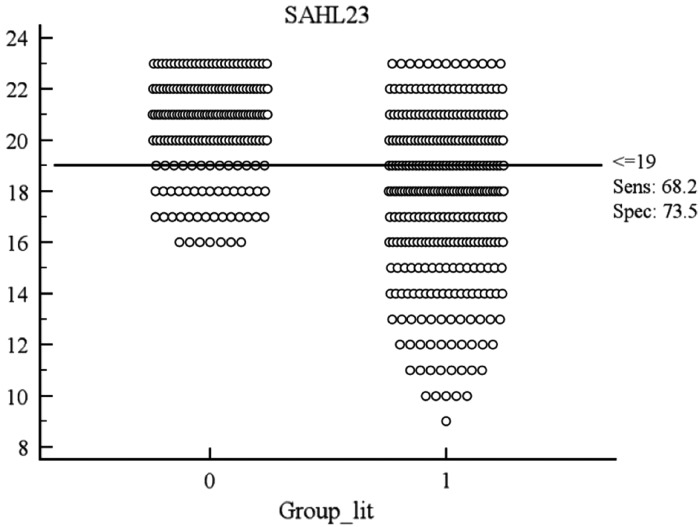
Interactive dot diagram with sensitivity and specificity plotted against the criterion value (*n*=484). SAHL=Short Assessment of Health Literacy.

## Footnotes

1
Cut-off value (<20) on the comprehension of PLs assessment (maximum score=24) was used to categorise participants into groups on the basis of health literacy: adequate (*n*=193; 38.4%) and inadequate (*n*=310; 61.6%).

2
Law – *Decreto-Lei* no. 42994/1960 and 85/2009.

## Appendix 1

**Table A1:** Template of the questionnaire

Numbers	Question
1. Quest A	What is the name of the medicine?
2. Quest B	What is the name of the principal component of the medicine?/What are the names of the principal components of the medicine?
3. Quest A	Imagine that you had <… >.^a^ How many/How much tablets/capsules/solution etc. can you take/apply per day/week, etc.?
4. Quest B	Imagine that you had <… >.^a^ How many times per day may you take apply this medicine?
5. Quest A	Imagine that you have a family member pregnant. Explain if this person may take/administer this medicine
6. Quest B	Imagine that you have a family member breast-feeding. Explain if this person may take/administer this medicine
7. Quest A	Imagine that you took too much medicine. Explain what you will do?
8. Quest B	Imagine that you forgot to take/administer the medicine. Explain what you will do?
9. Quest A	Imagine that you have <…>.^b^ Please indicate if you may take/administer this medicine
10. Quest B	Imagine that you were taking/applying another medicine with the following composition: <… >.^c^ Explain what may happen if you take/use these two medicines at the same time
11. Quest A	Explain if you may drive in case you are taking/administering this medicine
12. Quest B	Explain how you would take/administer this medicine
13. Quest A	Explain what is the action/effect of this medicine
14. Quest B	Explain how you would store this medicine
15. Quest A	What is/are the name(s) of the diseases/health problems that this medicine help(s) to treat/cure?
16. Quest B	Imagine that after you taking/applying the medicine appear <…>^d^ during several days. Imagine that after you taking/applying the medicine appear <…>^b^ during several days. Explain what you would do in case of this happen

Quest=questionnaire.

a A name of a disease was randomly selected based on the indications of the medicine described in the evaluated package leaflet (PL). The first name was always excluded because it was considered to be too accessible to the reader.

b A clinical situation related to a contraindication was randomly selected from the list in the PL (if the medicine did not present contraindications, one of the precautions listed in the PL was selected instead). The first contraindication/precaution was excluded because it was considered too accessible to the reader.

c The name of an active component that interacts with the medicine listed in the PL was randomly selected. The first interaction described in the text was excluded, because it was considered too accessible to the reader.

d A drug adverse reaction listed in the PL was randomly selected. The first drug adverse reaction described in the text was excluded, because it was considered too accessible to the reader.

## Appendix 2

**Table A2:** Scree plot for the SAHLPA-23 and SAHLPA-18

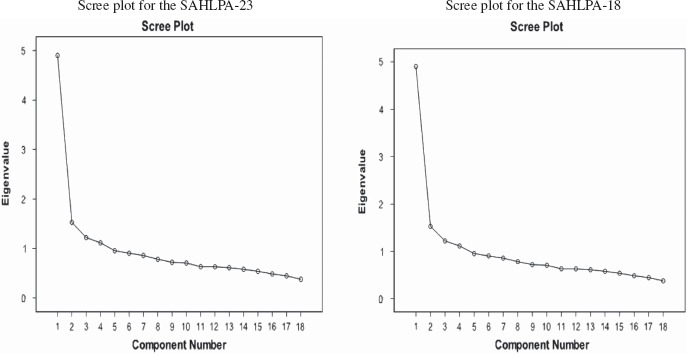

SAHLPA-18 and SAHLPA-23=Short Assessment of Health Literacy for Portuguese-Speaking Adults with 18 and 23 items, respectively. SAHLPA-23 was the only administered tool, that is, SAHLPA-18 is referent to a separate analyse comprising the results for the items of the original version of Apolinario *et al*. (2012).

## Appendix 3

Item-total statistics for SAHLPA-23 and SAHLPA-18

**Table A3:** Item-total statistics for SAHLPA-23

Items	Corrected item-total correlation	Cronbach’s *α* value if item deleted
Item 1: Prostate	0.282	0.708
Item 2: Active substance	0.457	0.689
Item 3: Drug adverse reactions	0.118	0.720
Item 4: Menstrual	0.279	0.710
Item 5: Caffeine	0.298	0.709
Item 6: Osteoporosis	0.188	0.714
Item 7: Incest	0.348	0.701
Item 8: Testicle	0.292	0.709
Item 9: Rectal	0.334	0.703
Item 10: Abnormal	0.304	0.708
Item 11: Miscarriage	0.141	0.717
Item 12: Excipient	0.301	0.708
Item 13: Jaundice	0.358	0.701
Item 14: Pap	0.375	0.699
Item 15: Appendix	0.100	0.719
Item 16: Fertility	0.233	0.712
Item 17: Behaviour	0.389	0.699
Item 18: Hemorrhoid	0.211	0.713
Item 19: Colitis	0.115	0.728
Item 20: Precautions	0.202	0.714
Item 21: Gallbladder	0.330	0.704
Item 22: Seizure	0.129	0.717
Item 23: Arthritis	0.406	0.698

SAHLPA-18 and SAHLPA-23=Short Assessment of Health Literacy for Portuguese-Speaking Adults with 18 and 23 items, respectively. SAHLPA-23 was the only administered tool, that is, SAHLPA-18 is referent to a separate analyse comprising the results for the items of the original version of Apolinario *et al*. (2012).

**Table A4:** Item-total statistics for SAHLPA-18

Items	Corrected item-total correlation	Cronbach’s α value if item deleted
Item 1: Prostate	0.349	0.807
Item 2: Menstrual	0.543	0.798
Item 3: Caffeine	0.478	0.801
Item 4: Osteoporosis	0.420	0.804
Item 5: Incest	0.379	0.806
Item 6: Testicle	0.457	0.802
Item 7: Rectal	0.429	0.801
Item 8: Abnormal	0.521	0.798
Item 9: Miscarriage	0.442	.804
Item 10: Jaundice	0.384	0.808
Item 11: Pap	0.353	0.808
Item 12: Appendix	0.363	0.806
Item 13: Behaviour	0.479	0.798
Item 14: Hemorrhoid	0.426	0.802
Item 15: Colitis	0.268	0.816
Item 16: Gallbladder	0.386	0.804
Item 17: Seizure	0.415	0.804
Item 18: Arthritis	0.481	0.798

SAHLPA-18 and SAHLPA-23=Short Assessment of Health Literacy for Portuguese-Speaking Adults with 18 and 23 items, respectively. SAHLPA-23 was the only administered tool, that is, SAHLPA-18 is referent to a separate analyse comprising the results for the items of the original version of Apolinario *et al*. (2012).
